# Obituary

**Published:** 1910-03-05

**Authors:** 


### Obituary.

The death is reported in London of Major A. L. Borradaile, R.A.M.C., retired, at the age of forty-five, of Langdon, Alta, Canada. Major Alfred Latour Borradaile studied at Edinburgh University, where he took the degrees of M.B. and C.M. in 1886.

The death is reported of Dr. Augustin Le Rossignol, Jurat of the Royal Court of Jersey, at the age of 68. He studied at Aberdeen University and the London Hospital, taking his M.B. and C.M. at the former in 1867, and his M.D. in 1868. He also gained his diploma in the same years. His appointments included the post of hon. consulting surgeon to the Jersey General Dispensary, and assistant resident medical officer at the London Hospital. He was also an ex-president of the Jersey Medical Society and late inspector-general of the medical staff of the Royal Jersey Militia.

